# Failure to scale in digital agronomy: An analysis of site-specific nutrient management decision-support tools in developing countries

**DOI:** 10.1016/j.compag.2023.108060

**Published:** 2023-09

**Authors:** Tesfaye Shiferaw Sida, Samuel Gameda, Jordan Chamberlin, Jens A. Andersson, Mezegebu Getnet, Lennart Woltering, Peter Craufurd

**Affiliations:** a International Maize and Wheat Improvement Center (CIMMYT), Addis Ababa, Ethiopia; b International Maize and Wheat Improvement Center (CIMMYT), Nairobi, Kenya; c Plant Production Systems, Wageningen University and Research (WUR), Wageningen, the Netherlands; d Stichting Wageningen Research (SWR), Addis Ababa, Ethiopia; e International Maize and Wheat Improvement Center (CIMMYT), El Batán, Texcoco, Mexico; f International Maize and Wheat Improvement Centre (CIMMYT), Kathmandu, Nepal

**Keywords:** Fertilizer recommendation, Agronomy at scale, Precision agriculture, Digital agriculture, Scalability

## Abstract

While many have extolled the potential impacts of digital advisory services for smallholder agriculture, the evidence for sustained uptake of such tools remains limited. This paper utilizes a survey of tool developers and researchers, as well as a systematic *meta*-analysis of prior studies, to assess the extent and challenges of scaling decision support tools for site-specific soil nutrient management (SSNM-DST) across smallholder farming systems, where “scaling” is defined as a significant increase in tool usage beyond pilot levels. Our evaluation draws on relevant literature, expert opinion and apps available in different repositories. Despite their acclaimed yield benefits, we find that SSNM-DST have struggled to reach scale over the last few decades and, with strong heterogeneity in adoption among intended stakeholders and tools. For example, the log odds of a SSNM-DST reaching 5–10 % of the target farmers compared with reaching none, decreases by ∼200% when a technical problem is stated as a reason for the tools’ failure to be used at scale. We find a similar decrease in odds ratios when technical, socioeconomic, policy, and R&D constraints were identified as barriers to scaling by national extension and private systems. Meta-regression analysis indicates that the response ratio of using SSNM-DST over Farmer Fertilizer Practice (FFP) varies by non-tool related covariates, such as initial crop yield potential under FFP, current and past crop types, acidity class of the soil, temperature and rainfall regimes, and the amount of input under FFP. In general, the SSNM-DST have moved one step forward compared with the traditional ‘blanket’ fertilizer recommendation by accounting for in-field heterogeneities in soil and crop characteristics, while remaining undifferentiated in terms of demographic and socioeconomic heterogeneities among users, which potentially constrains adoption at scale. The SSNM-DSTs possess reasonable applicability and can be labeled ‘ready’ from purely scientific viewpoints, although their readiness for system-level uptake at scale remains limited, especially where socio-technical and institutional constraints are prevalent.

## Highlights

• We appraise evidence on the uptake of decision support tools for site-specific nutrient management (SSNM-DSTs).
• SSNM-DSTs still struggle to reach scale, despite evidence of benefits.
• Scaling constraints vary by type of tool and user characteristics.
• SSNM-DST address in-field heterogeneity, but not socioeconomic heterogeneity of users.
• Future work should include institutional, economic and governance aspects of scaling.

## 1 Introduction

Innovations in digital technology have benefited many scientific disciplines and economic sectors, including agriculture. This can be evidenced by the boom of initiatives such as Digital Earth (Guo, 1998), Digital Agriculture (Tang et al., 2002), Precision Agriculture (Cassman, 1999, Gebbers and Adamchuk, 2010, Zhang et al., 2002), Virtue Agriculture (Tang et al., 2002), Information Agriculture (Hornik, 1993), Smart Agriculture/Farming (Janc et al., 2019, Knierim et al., 2019) and Digital Farming (Bronson, 2019) since the 1990s. Digital agriculture, which is the application of digital tools and technologies in agriculture, offers multiple potential benefits to smallholders. First, it can assist in addressing the problem of location-specific yield gaps through the optimum allocation of mineral and organic fertilizers (Jat et al., 2013). Second, it can help to reduce the environmental impacts of agriculture – nutrient leaching and greenhouse gas emissions – by matching nutrient inputs to plant nutrient requirements (Deichmann et al., 2016, Liang et al., 2013). Third, it allows the collection of site-specific biophysical and management data (Basso and Antle, 2020), with a potential to enhance consequent data-driven decision-making in smallholder systems (Chandra and Collis, 2021).

Globally, digital agriculture has been applied, albeit with varying degrees of success, to guide fertilizer recommendations (MacCarthy et al., 2018), operationalize ecosystem services (MacCarthy et al., 2018), optimize soil nutrient management (Pooniya et al., 2015, Sapkota et al., 2021, Singh, 2019), understand trade-offs in climate-smart agriculture (Capalbo et al., 2018), adjust irrigation management (Wellens et al., 2017) and analyze the effect of climate change on inland fisheries (Lynch et al., 2015). We limit the current analysis to its application in the form of Site-Specific Nutrient Management Decision Support Tools (SSNM-DST). In the smallholder farming systems that are prevalent in developing countries, SSNM-DST have been applied predominantly in soil nutrient management (AfricaRice, 2014, Buresh and Witt, 2007, Johnston et al., 2009, Kaizzi et al., 2013, Prause et al., 2021). The concept of site-specific nutrient management integrates information from different scales to assist field-specific decisions (Chivenge et al., 2022, Pampolino et al., 2012). The greater success of SSNM-DST over farmers’ fertilizer practices (FFP) is measured through improved yields (Bhatta et al., 2020, Chuan et al., 2013, Jansen et al., 2013, Pampolino et al., 2012, Rurinda et al., 2020, Saito et al., 2015, Xu et al., 2016), higher returns (Bhatta et al., 2020, Jansen et al., 2013, Pampolino et al., 2012, Saito et al., 2015, Zhang et al., 2018), and better environmental quality (Wang et al., 2020, Zhang et al., 2018) as a result of tailored advice provided by the tools. In Africa alone, nearly 400 different digital agriculture solutions have been on the market (World Bank Group, 2019). Nonetheless, the solutions reached only 6 % of an estimated €2.3 billion potential advisory market in the continent (Tsan et al., 2019).

Uptake of SSNM-DST at scale in smallholder farming systems has been limited (Shepherd et al., 2020) despite claims of its numerous co-benefits (Chivenge et al., 2021) and in contrast to the fast rate of expansion of digital technologies in other sectors (Bhavnani et al., 2016, Goggin, 2006, Kaur et al., 2021, Mallat et al., 2004, Topol, 2019). One explanation could be the absence of combined efforts among technology developers, agronomists, socio-economists, behavior experts, tool designers, political economy experts, as well as the lack of involvement of scaling partners as has been found to be the case in other sectors (Woltering et al., 2019). Often times, algorithm developers in SSNM-DST do not have the required data and computational tools needed to convert intricate soil and plant geospatial information into appropriate crop management actions (Capalbo et al., 2018). Consequently, the SSNM-DST appear to suffer from incomplete understanding and misuse by end users, mainly agronomists and extension workers (Andersson et al., 2020), leading to limited chances for adoption at scale (Ayim et al., 2022). Furthermore, SSNM-DST are piloted in controlled project environments – i.e., in trials, evaluations or pilots – that do not reflect the realities of smallholders (Woltering et al., 2019). Another mentionable limitation is that SSNM-DST already on the market have been calibrated under data-scarce settings (Shepherd et al., 2020). There have been complaints about the tools being rigid and knowledge intensive (Andersson et al., 2020), making them difficult to be adapted to smallholder conditions. Most tools lack utilities that allow inclusion of site-specific soil and agronomic information while generating advice. Yet, they have been massively promoted in many countries of Sub-Saharan Africa (SSA), South Asia and Southeast Asia, although the level of adoption at scale for these tools has not been well documented.

‘Blanket’ fertilizer advisories remain the main approach of choice in many places (Tefera et al., 2020), regardless of the large inter- and intra-plot heterogeneities inherent in smallholder fields (Sida et al., 2021), underscoring the importance of site-specific approaches. Such systems suffer from low fertilizer use efficiency, low factor productivity and persistent food insecurity (Aleminew and Alemayehu, 2020). Digital agriculture has been established rapidly in large-scale, capital intensive agriculture (Kelley et al., 2020, Lindblom et al., 2017, Tang et al., 2002), although the drivers of adoption have not been firmly established, even in those systems (Nowak, 2021, Tey and Brindal, 2022). Although not at a similar rate to developed economies (DeGusta, 2012), accessibility of digital technology is destined to improve in developing countries (DeGusta, 2012, Duncombe, 2016). The recent explosion of mobile phone technologies and smallholders’ relative ease of access to smartphone-based applications offer an opportunity to advance digital agronomy in smallholder systems. Nonetheless, drivers of (non)adoption, especially at the regional level, have never been explored.

The current work aims to explore the level of scaling of SSNM-DST within smallholder farming systems, where we define “scaling“ as a significant increase in tool usage beyond pilot levels. We aim to highlight why these tools have failed to be used at scale, regardless of their potential benefits. We combine survey and *meta*-analysis hybrid methods and seek to answer the following questions regarding the application of digital site-specific nutrient management decision-support tools.

(1) How prominent are digital advisory tools for site-specific nutrient management and what is their current level of adoption in smallholder farming systems?
(2) What are the main drivers of (non)adoption of site-specific nutrient management decision support tools under smallholder contexts?

## 2 Materials and methods

### 2.1 Literature search and tool identification

We made an inventory of site-specific nutrient management decision support tools (SSNM-DST) that are available (or under development) within the context of smallholder farming. We conducted two rounds of systematic literature search.

In the first round, we searched major academic search engines Google Scholar, Scopus and ISI Web of Science for documents that reported about site-specific nutrient management decision support tools, using the search strings [“decision support tool*” AND “site-specific nutrient management”]. We limited our search to the period from 2000 to 2020. We focused on this period because the concept of site-specific nutrient management started to emerge in the 1990’s (Dobermann and White, 1999, Reetz and Fixen, 1995) and use of decision support tools emerged slightly later (Betteridge, 2006). The literature turnout corroborates our assumption, as it shows that trials exploring SSNM-DST started to emerge in the late 1990’s and publication of their results started to emerge since the early 2000’s. It is important to note that we focused only on peer-reviewed literature, excluding any gray literature on this topic. This is because we were interested in only including manuscripts with robust study designs and that had passed through a rigorous revision process. However, we acknowledge that such parsimonious selections may downwardly bias the number of studies included in our analysis and potentially upwardly bias the types of studies that reject the null hypothesis, which arises from selective publication of positive results. The latter scenario is in line with our objectives since we are interested in understanding the challenges of scaling SSNM-DST, even when they are proven to be profitable (i.e., resulting in positive outcomes). The search at this round returned 379 papers in total, reporting on the results of seven SSNM-DST (Table 1).

**Table 1:** List of site-specific nutrient management decision support tools (SSNM-DST) selected from the literature.

SSNM-DST Full Name		SSNM-DST Abbreviation		Descriptions		Countries* of implementation		Back-end Model		Primary users		Media	
Fertilizer optimization tool		FOT		FOT takes input information on crops, acreage, projected price of crop at the time of harvest, cost of fertilizer and the amount of money to be invested by the farmer are required. Fertilizers to be applied to each crop, its expected mean effect on yield, net returns for each crop, and the expected total net returns are the outcomes. The tool recommends fertilizer rates that optimizes return on investment based on predetermined nutrient response curves.		Burkina Faso, Ethiopia, Ghana, Kenya, Mali, Mozambique, Niger, Nigeria, Rwanda, Tanzania, Uganda, Zambia		Excel Solver ®		Extension agents & Farmers		PC, Paper, App	
Generic		Generic		These include tools that explicitly mention site-specific decision and follow the principles of Site-Specific Nutrient management decision-support tools but cannot fully fall under a specific tool. For example, tools that use target yields and simulation modelling in combination with site specific soil test results were included under this category.		Bangladesh, Burkina Faso, China, Ethiopia, India, Indonesia, Nepal, Philippines, Senegal, Thailand, Vietnam		NA		Farmers, extension, private sectors, development and, research institutes		Multiple	
Leaf Colour Chart		LCC		LCC had been jointly developed by International Rice Research Institute (IRRI) and Philippines Rice Research Institute (PhilRice) from a Japanese prototype, for the purpose of measuring the required quantity of nitrogen to be applied in crop fields, leading to maximum productivity. The LCC has 4–6 green strips, with colour ranging from yellow green to dark green. It determines the greenness of the rice leaf, which indicates its N content.		Bangladesh, China, India, Indonesia, Nepal, Philippines, Thailand, Vietnam		NA		Extension, farmers		Digital/ Physical Gadget	
Nutrient Expert®		NE		NE estimates the attainable yield and yield response to fertilizer from site information using decision rules developed from on-farm omission trials. It uses characteristics of the growing environment: water availability, soil health and fertility indicators, historical use of organic materials, crop sequence, crop residue management, fertilizer input and crop yields for the previous season.		Bangladesh, China, Ethiopia, India, Indonesia, Nepal, Nigeria, Philippines, Vietnam,		QUEFTS		Extension agents		PC, Web, App	
Rice Crop Manager		RCM		RCM is designed for use by extension workers, crop advisors, agricultural service providers, and farmer leaders. It uses farmer's answers to questions on rice farming practices to automatically generate crop management guidelines aimed at increasing the net income.		Bangladesh, India, Indonesia, Myanmar, Philippines, Vietnam		Oryza		Extension workers, crop advisors, input providers, service providers		Web	
RiceAdvice		RiceAdvice		RiceAdvice is a decision support tool developed by the International Rice Research Institute (IRRI) and Africa Rice Center (AfricaRice) for rice crop management. It aims at providing smallholder rice-farmers with timely field specific guidelines for crop and nutrient management practices. The guidelines are generated each new season.		Burkina Faso, Cameroon, Chad, Côte d'Ivoire, Ethiopia, Ghana, Madagascar, Nigeria, Senegal, Sierra Leone, Togo		Oryza		Farmers, extension agents, private sectors, development agencies		App	
The Soil Plant Analysis Development (SPAD)		SPAD		Chlorophyll meter is used to generate relative values of leaf chlorophyll content, which is used as an indicator of the health status of crops and guide for crop fertilization and field management in different crop growth periods. The SPAD values are the ratio of the amount of incident infrared to the emitted infrared radiation (IR) to the ratio of incident red to emitted red wavelengths (R) of the visible spectrum. The value indicates the greenness level of leaves, which in turn shows the level of leaf N content, guiding application of N-containing fertilizer at different crop growth stages.		Bangladesh, China, India, Indonesia, Mozambique, Nepal, Philippines, Vietnam		NA		Extension agents, Farmers		Digital Gadget	

*The table lists only countries where the tools in the current analysis have applied in.

**Fig. 1:**
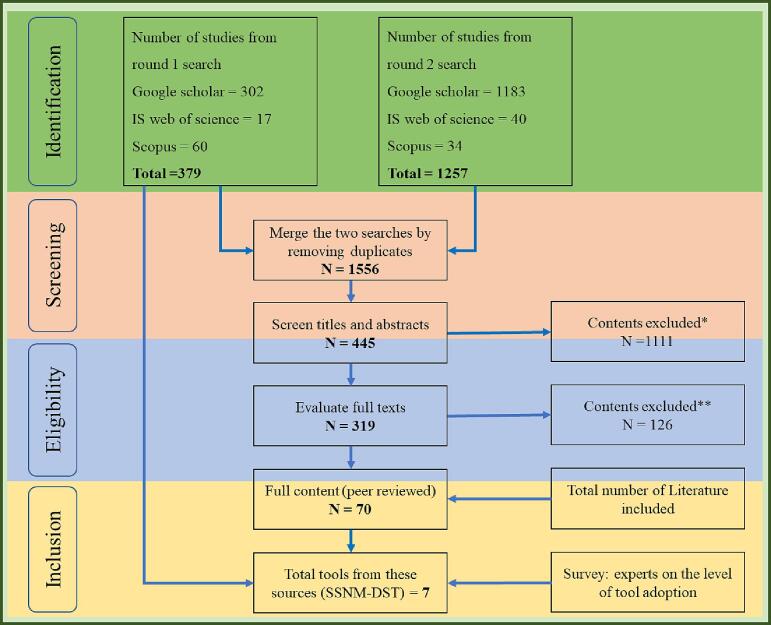
Workflow showing the selection procedure of SSNM-DST for evaluation for inclusion in the appraisal. Superscripts a and b, respectively, indicate the application of the first and second screening criteria explained in the text. Seventy published sources with a total of 442 entries were included for the seven SSNM-DST explored.

In round two, we read the abstracts and scanned through each paper to identify the exact name of the SSNM-DST as compiled in Table 1. We then used these names as search terms to undertake more rounds of literature revision. We followed this stage to avoid any potential bias that may have occurred if we only selected studies that included the SSNM-DST by its name (e.g., Nutrient Expert) without explicitly mentioning the search terms ‘site-specific nutrient management’ or ‘decision support tool’ used in the earlier round. The tool-based searches identified 1257 documents. It is apparent that some SSNM-DST may not have been fully captured using the initial search. We merged this with results from the previous search, retaining a total of 1556 documents for further screening (Fig. 1). We removed duplicates and documented the papers for further screening using pre-set exclusion criteria.

Following the screening workflow summarized in Fig. 1, we first screened the titles and the abstracts of the documents and excluded: (1) documents that were not dealing with any form of decision support system in soil nutrient management and (2) documents that discussed decision support tools, but that were not applied to smallholder systems. We retained 445 documents based on these criteria. Second, we explored the full texts of the retained documents and excluded irrelevant literature. The criteria for eligibility were: (1) the tool served farmers or service providers to assist in soil fertility management; (2) The developer clearly stated that the tool was for site-specific nutrient advisory purposes; (3) the tool serves to either optimize nutrient use efficiency or economic benefits from mineral and organic fertilizers; (4) The tool has been applied at least at performance trial levels; and (5) The study clearly compared the benefit of using SSNM-DST with Farmer Fertilizer Practices that are commonly recommended for the region of interest (FFP). Under conditions where both FFP and local recommendations were included in the studies, we selected the FFP for our analysis. We excluded studies where agronomic management or factors other than nutrient management differed between FFP and SSNM-DST plots. A total of 70 documents with 442 entries were included in our study. We summarize the details of the literature included for analysis in Table S1 (https://doi.org/10.7910/DVN/GRYA0U) and the stages in Table S2 (https://doi.org/10.7910/DVN/I7U8YY).

### 2.2 Data compilation

We collected a variety of variables from the studies listed in Table S1, including details about the study location, measurement parameters, and study design. Additionally, we included information about the specific crops grown and the SSNM-DST utilized for soil nutrient management. Measured outcomes such as yield, nutrient use efficiency, and environmental impacts resulting from the tool application were quantified, along with corresponding values reported for FFP. When studies involved multiple treatment levels, locations, and SSNM-DST variations for a particular trial, we recorded data separately for all tools, crops, and sites. In cases where results were not presented in tabular form and were challenging to extract directly, we utilized the metaDigitise package in R to extract values from graph-based summary reports (Pick et al., 2019).

In addition to measured values, we compiled data on potential covariates for the experimental plots, encompassing environmental factors (e.g., region, rainfall, temperature regime, soil properties), management practices (e.g., current and past crops, tillage methods), and input variables (such as the type and application rate of fertilizer, fertilizer cost).

### 2.3 Survey of current level of tool adoption

We acknowledge that identifying the exact stage and level of adoption for a technology is challenging. Consequently, we emphasize that our survey results reflect the views of tool developers, which may not always align with perspectives expressed by tool users. To appraise the current stage of adoption for the tools in Table 1, we compiled the email addresses of corresponding authors and co-authors from the literature identified in Table S1. Additionally, we created mailing lists of institutions, national systems, consortiums, and individuals working with the identified SSNM-DST.

We then sent a questionnaire with customized messages to each stakeholder, addressing various aspects of the SSNM-DST, such as its type, application location, proportion of target farmers currently using the tool, uptake by the national extension system and/or the private sector, stage of adoption within the target region, and potential drivers for adoption trends. Approximately 414 customized emails were circulated (exact receiver count unknown due to inclusion of specialist group mailing lists with unidentified list sizes). Following three months of open survey with monthly reminders, we received 81 responses, which were used for analysis. While the response rate was approximately 20%, the survey results remain informative since the respondents were specialists on SSNM-DST. The number of experts per tool ranged from six (RiceAdvice) to 21 (NE), with an average response rate of 16 per tool.

### 2.4 Data analysis

#### 2.4.1 Analysis of survey data

To analyze the adoption levels of SSNM-DST among various stakeholders, we employed multinomial logistic regression. We derived the dependent and independent variables for tool adoption from survey responses. The dependent variable in this study represents three adoption categories for farmers: <5% coverage (considered as no adoption, also used as the baseline), 5–10% adoption, and more than 10% adoption. Similarly, three adoption categories were defined for both national extension and private business systems: no adoption, partial adoption, and full adoption. Since all variables are categorical, we applied multinomial logistic regression (MNL) to model them. The variables identified as drivers of adoption level for all stakeholder groups (farmers, extension, and business) were classified into four categories: technical, socioeconomic, policy, and R&D. Technical variables encompassed aspects such as tool complexity, time requirement, required education level, and data needs. Socioeconomic variables included factors like phone access, telecom network coverage, farmer behavior, and extension-to-farmer ratio. Policy variables pertained to government strategy, extension services, advocacy level, and capacity building activities. R&D variables focused on proper calibration, tool consistency, stability, scaling readiness testing, and scaling approach.

To estimate the multinomial logistic regression model, we employed the 'multinom' function from the 'nnet' package in R (Ripley et al., 2016). We fitted separate regressions for each stakeholder category using the logistic regression of the form presented in Eq. (1). The regression outcome facilitated the identification of the specific contributions of technical, socioeconomic, business model, and policy aspects in constraining the tool's adoption at scale.

(1) $$ln\left(\frac{P\left(\chi_{o}\right)}{P\left(\chi_{i}\right)}\right)=\beta_{0}+\beta_{1}\tau_{i}+\beta_{2}\phi_{i}+\beta_{3}\rho_{i}+\beta_{4}\psi_{i}+\varepsilon_{i}$$

where, $P\left(\chi_{o}\right)is$ the probability of choosing the baseline (non-adoption) category, $P\left(\chi_{i}\right)$ is the i^th^ probability of choosing an outcome category other than the baseline category, $\tau_{i}$, $\phi_{i}$, $\rho_{i}$, and $\psi_{i}$ are the i^th^ responses in the outcome categories for technical, socioeconomic, policy and R&D variables, respectively, $\beta_{0}$ is the constant term of the regression, $\beta_{1},\beta_{2,}\beta_{3}and\beta_{4}$ are regression coefficients for the respective variables in the outcome category and $\varepsilon_{i}$ is the error term of the regression. We used the probability level of 0.05 to assess the significance of each effect size in the model, unless otherwise stated.

#### 2.4.2 Analysis of review data

Proportions of negative, neutral and positive yield, economic and environmental effects of using a SSNM-DST over the FFP were computed using Eq. (2).

(2) $$\delta Y_{i}=\left(\frac{Y_{i(SSNM-DST)}-Y_{i(FFP)}}{Y_{i(FFP)}}\right)*100$$

where $\delta Y_{i}$ denotes the relative change in effect for the i^th^ SSNM-DST, $Y_{i(SSNM-DST)}$ is the yield, economic and environmental values reported under the i^th^ SSNM-DST, and $Y_{i(FFP)}$ is the value of these effects reported for a corresponding FFP. To assess the economic impact, we employed the marginal rate of return (MRR) on fertilizer investment. We evaluated the environmental impact by determining the partial factor productivity (PFP) of nitrogen fertilizer, which measures kilogram of grain yield obtained per kilogram of applied nitrogen. We considered lower values of grain yield per kilogram of nitrogen as a proxy for negative environmental effects. We categorized the values of $\delta Y_{i}$ as negative, neutral, or positive. Relative change values between −5% and 5% from the corresponding FFP values were labeled as neutral. Values below −5% were considered negative, and values above 5% were considered positive. For the PFP of nitrogen fertilizer, we used a reversed rating where values lower than −5% were considered positive, indicating a reduction in negative environmental consequences. Using this approach, we summarized the proportions of negative, neutral, and positive relative changes for each analyzed SSNM-DST in this study.

In addition to exploring the drivers of adoption (or lack thereof) through the survey questionnaire, we hypothesize that variables aggregated from the *meta*-analysis results can reveal some drivers of scaling. Therefore, we utilized a random-effect model to determine the impact of using the tool-assisted strategy on crop yield under SSNM-DST compared with FFP. We calculated the effect size as the natural log of the response ratio (RR) between the two yields (Hedges et al., 1999), representing the effects of using the tools over FFP Eq. (3).

(3) $$ln\left(RR\right)=ln\left(\frac{Y_{T}}{Y_{F}}\right)$$

where RR is the response ratio, $Y_{T}$ and $Y_{F}$ the crop yield, using SSNM-DST tools and FFP, respectively. A *meta*-regression was conducted modeling the RR as the dependent variable. The effect of using SSNM-DST in soil nutrient management was explored by controlling for all the other covariates relating to the experimental environment (e.g., region, rainfall, temperature regime, soil properties), management (e.g., current crop and past crops, tillage practices) and inputs (type and rate of fertilizer applied, cost of fertilizer). Due to inevitable experimental heterogeneity, we combined data using a random-effects model applied to sub-grouped covariates and conducted subgroup analyses to unravel the confounding effects of experimental environment, management, input and geographic features on the effect size of the response ratio. We identified the most important covariates using the AIC forward elimination using the 'olsrr' package in R (Hebbali and Hebbali, 2017) and performed *meta*-analysis using the ‘metareg’ function of the ‘meta’ package in R (Schwarzer, 2007) with the selected covariates. We conducted *meta*-regression analysis following the general model outlined in Eq. (4).

(4) $${\hat{\theta }}_{i}=\theta +\;{\beta \chi }_{i}+\;\varepsilon_{i}+\;\omega_{i}$$

where ${\hat{\theta }}_{i}$ is the effect size of study i, θ is the fixed term and $\chi_{i}$ is a vector of predictors (covariates) with β being a vector of regression coefficients. Unlike the conventional random-effects-model, the meta regression has two error terms (i.e., $\varepsilon_{i}\;and\;\omega_{i}$), where $\varepsilon_{i}$ is the sampling error through which the effect size of a study deviates from its true effect and $\omega_{i}$ is the true effect size of the study that is only sampled from an overarching distribution of effect sizes.

In addition, the size of the response ratio as a function of crop yield under FFP was assessed and visualized using quantile regression fitted to the 90th percentile of the pooled data with the ‘rq’ function of the ‘quantreg’ package in R (Koenker et al., 2018). The relationship was assumed to take a logistic functional form (y = a - b × x + c × 0.90^x^), where y refers to the natural log of RR, x to the yield under FFP and a, b and c to the instantaneous slopes (first degree derivatives) of the quantile regression curves.

## 3 Results

### 3.1 Trends in the application of SSNM-DST and their comparative advantages

**Fig. 2:**
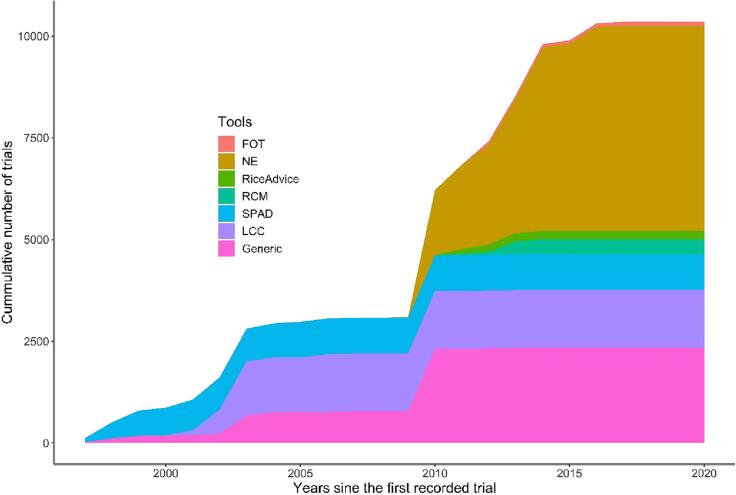
Trends in the number of trials with digital SSNM-DST. For the explanation of the abbreviations, refer to Table 1.

The popularity of SSNM-DST has been consistently increasing over the past 20 years, as shown by the growing number of experiments involving these tools (see Fig. 2). However, there has been a clear shift in the focus towards specific types of tools. Until the early 2010s, the dominant tools were Soil Plant Analysis Development (SPAD), Leaf Color Chart (LCC), and Generic tools. These tools were gadgets that helped make on-site decisions based on crop characteristics but were not fully developed into digital applications. Nutrient Expert (NE) appears to be the first tool to apply app-based advisory for site-specific nutrient management, and it became the most popular among SSNM-DST starting from the late 2000s. Other app-based tools like Fertilizer Optimization Tool (FOT), RiceAdvice, and Rice Crop Manager (RCM) emerged in the early to mid 2010s. Currently, the app-based tools are gaining popularity, while the earlier gadget-based tools are declining.

Although the majority of the SSNM-DST have positive to neutral effects, there are some negative effects when comparing their application to traditional FFP. In terms of yield, the overall negative effect is only about 5%, with the biggest proportion of negative effect found in SPAD (15%). When considering the economic effect, measured by the marginal rate of return on investment, RiceAdvice shows the largest negative effect at around 14%. Overall, the negative effect for this indicator stands at 3.2%. SSNM-DST generally have few negative effects on environmental outcomes, with an overall negative effect of about 18% and overall positive environmental outcomes of over 70%. RCM has the largest negative environmental consequence, with 50% of the advisory incidences resulting in lower grain yield per kilogram of applied nitrogen (Table 2).

**Table 2:** Proportion of negative, neutral and positive yield, economic and environmental effects for each of the SSNM-DST included in the current analysis. For every effect (yield, economic and environmental), values between −5 disadvantage and 5 % advantage of using SSNM-DST over FFP were considered neutral, values that are more disadvantageous than −5 % were considered negative and values with advantages of more than 5 % were considered positive. Economic effects were derived form marginal rate of return on investment for fertilizer, while environmental effects were derived from PFP of N fertilizer. The full description of the outcome variables is presented in Table S1 (https://doi.org/10.7910/DVN/GRYA0U).

Outcomes	Tools used in SSNM decision support							Overall
	FOT	Generic	LCC	NE	RCM	RiceAdvice	SPAD	
Yield effects								
Negative	—	3.4%	—	5.3%	—	—	15.0%	4.8%
Neutral	69.2%	20.7%	18.7%	16.5%	20.0%	14.3%	26.7%	20.6%
Positive	30.8%	75.9%	81.3%	78.2%	80.0%	85.7%	58.3%	74.7%

Economic effects								
Negative	—	3.4%	2.2%	1.1%	—	14.3%	11.7%	3.2%
Neutral	100.0%	41.4%	30.8%	52.7%	24.0%	—	25.0%	41.9%
Positive	—	55.2%	67.0%	46.3%	76.0%	85.7%	63.3%	55.0%

Environmental effects*								
Negative	—	23.4%	21.3%	7.1%	50.0%	—	18.3%	17.9%
Neutral	—	10.6%	6.7%	15.2%	18.2%	—	6.7%	11.0%
Positive	—	66.6%	72.0%	77.7%	31.8%	100%	75.0%	71.2%

Number of entries (N)								
Yield	13	58	91	188	25	7	60	442
Economic	17	58	88	188	22	7	49	429
Environmental	9	43	36	123	23	7	42	283

* Note: Fore the environmental effect, we used kg of Nitrogen from fertilizer per kg of grain produced under the corresponding advisory, also called PFP of Nitrogen; less kg of grain for more kg of N assumed to serve as a proxy for negative environmental effects.

### 3.2 Extent of scaling for SSNM-DST in smallholder systems

**Fig. 3:**
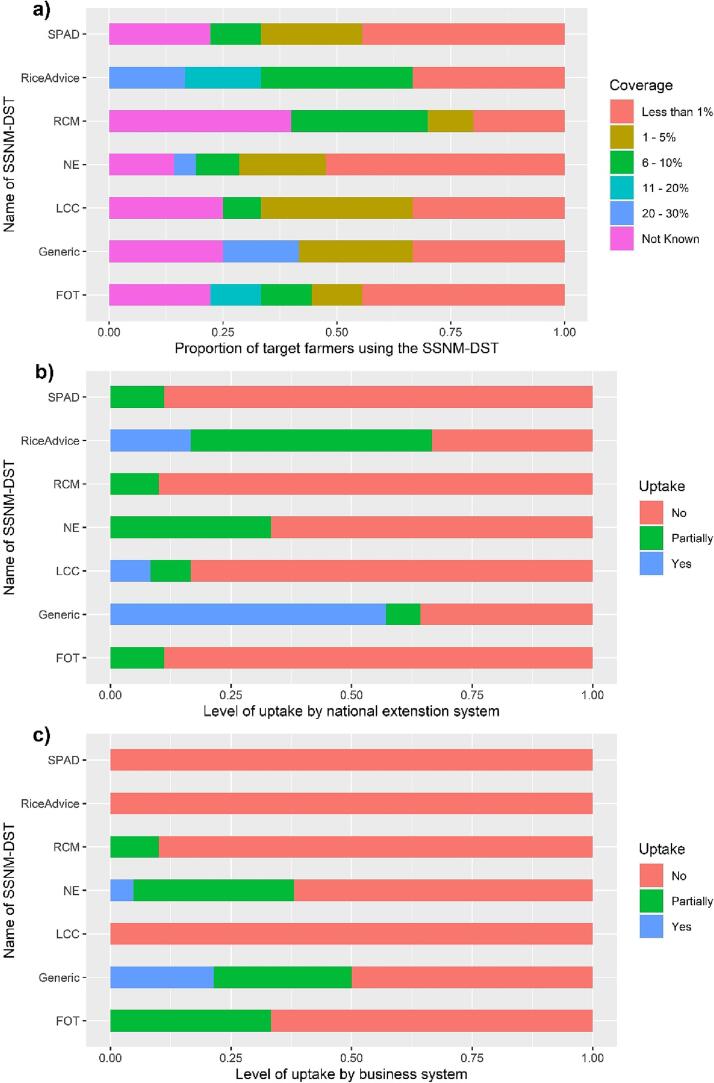
The reported level of adoption for each of the identified SSNM-DS tools by farmers (a), national extension system (b) and the private business (c). For the explanation of the abbreviations of the tools, refer to Table 1.

Despite growing interest and an expanding range of tools available (Fig. 2), the adoption of SSNM-DST has been limited (Fig. 3). There is variation in adoption levels among different tools and intended stakeholders. The most commonly adopted tools among farmers, such as generic tools, NE, and RiceAdvice, reached a maximum of only 20–30% of the target farmers (Fig. 3 a). However, <20% of respondents reported this adoption rate. Additionally, around 25% of responses indicated significantly poor adoption levels, with the tools reaching barely 1% of the target population. FOT had the lowest adoption rate, with nearly half of the respondents stating that it reached <1% of the target population.

The adoption levels of SSNM-DST by national extension systems (Fig. 3 b) and private businesses (Fig. 3 c) were even lower. RiceAdvice and generic tools were adopted or partially adopted by national extension systems approximately 65% of the time (Fig. 3 b). For the other tools, there was no evidence of adoption by national extension systems in over 75% of the cases (Fig. 3 b). NE was reported to be partially adopted by the extension system in about one-third of the cases. The uptake by private businesses was mostly non-existent for most tools (Fig. 3 c). Only two SSNM-DST (NE at 5% and generic at 20%) were reported to be taken up by the business system. Partial uptake of NE, FOT, and generic tools was indicated about 30% of the time, while partial uptake of RCM by private businesses was reported in about 10% of the cases. RiceAdvice, SPAD, and LCC were completely disregarded by the private system.

### 3.3 Challenges to scaling SSNM-DST: Expert accounts

Attempts to apply SSNM-DST at scale in smallholder systems posed different challenges across stakeholders (Table 3). When a technical problem was cited as the cause of the tools' failure to be used at scale (Table 3 panel I), the likelihood of at least 5% of target farmers adopting SSNM-DST was significantly low. Likewise, reporting a technical problem as a reason for the tools' failure to be used at scale resulted in a 177% decrease in the probability of SSNM-DST reaching more than 10% of the target farmers, compared to reaching none.

**Table 3:** Generalized logistic regression model results from the online survey data set. Dependent variables varied with the type of stakeholder (farmer, extension, and business). The question is whether a suggested reason is responsible for lack of uptake with baselines of uptake rate lower than 5% for farmers, no involvement at all for national extension and business systems. Values in the parentheses are the standard errors.

Stated reasons for no/low uptake of SSNM-DST									
		Technical		Socioeconomic		Policy		R&D	
Stakeholder	Level of SSNM-DST uptake	Parameterestimates	P-value	Parameterestimates	P-value	Parameterestimates	P-value	Parameterestimates	P-value
Farmer (I)	Intercept (<5% uptake)	−2.43 (1.37)	0.0772	−2.43 (1.37)	0.0772	−2.43 (1.37)	0.0772	−2.43 (1.37)	0.0772
	5–10% uptake	−1.85 (0.66)	**0.0052**	0.46 (0.96)	0.6334	−2.79 (1.52)	0.0661	2.65 (1.40)	0.0591
	>10% uptake	−1.77 (0.65)	**0.0067**	−0.87 (1.26)	0.4897	−0.18 (1.60)	0.9123	−13.22 (404.91)	0.9739
Extension (II)	Intercept (No uptake)	−17.27 (1.07)	**< 0.0001**	−1.17 (1.26)	**< 0.0001**	−17.27 (1.07)	**< 0.0001**	−1.17 (1.26)	**< 0.0001**
	Partial	−3.00 (1.02)	**0.0034**	1.29 (1.41)	0.3611	−17.61 (0.63)	**< 0.0001**	−17.36 (0.92)	**< 0.0001**
	Yes	−12.13 (91.6)	0.8947	–22.90 (0.01)	**< 0.0001**	0.29 (1.85)	0.8768	0.29 (1.85)	0.8769
									
Business (III)	Intercept (No uptake)	−1.10 (1.14)	0.0770	1.82 (1.27)	0.1687	−1.10 (1.14)	0.0770	1.82 (1.27)	0.1687
	Partial	−0.67 (1.20)	0.2902	−1.54 (1.64)	0.1730	1.59 (1.36)	0.1199	−0.62 (1.28)	0.3131
	Yes	−4.70 (1.55)	**0.0012**	0.05 (1.37)	0.4865	−1.60 (1.73)	0.1777	−1.15 (1.72)	0.2517
*Model fit parameters*									
	Log-Likelihood = –22.67								
	McFadden R^2^ = 0.30								
	chisq = 19.46								

The chances of the national extension system partially picking up a SSNM-DST decrease significantly when technical, policy, and R&D reasons are cited as the reasons for their lack of adoption (Table 3, panel II). Similarly, the likelihood of the extension system fully embracing the SSNM-DST compared to not adopting it at all decreases significantly when socioeconomic issues are mentioned as a factor hindering widespread tool adoption. Lastly, when technical problems are highlighted as the reason for poor tool adoption at scale, the likelihood of the private business system fully adopting the SSNM-DST compared to its failure to adopt it decreases significantly.

### 3.4 Challenges of scaling SSNM-DST: Dissecting published sources

**Fig. 4:**
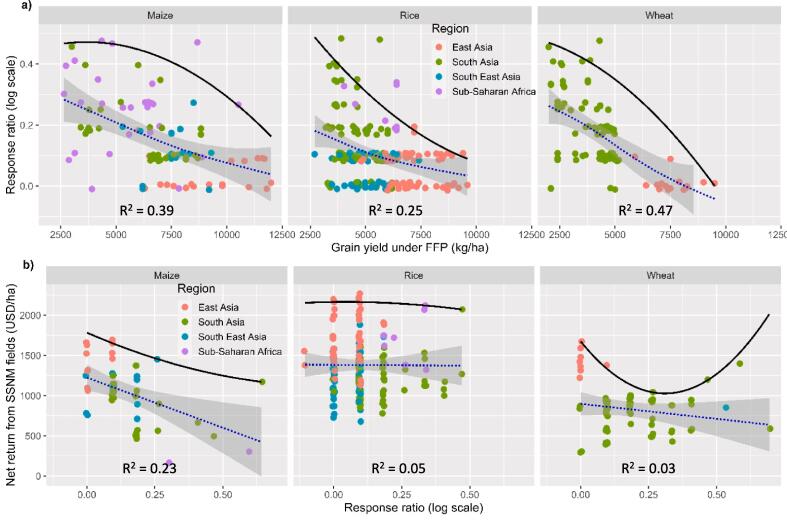
The relationship between response ratio of using SSNM-DST and grain yield across regions (a) and the relationship between the net return from SSNM fields and the response ratio (b) for the three crops (Maize, Rice and Wheat). Each observation corresponds to one individual study in each of the four regions. The solid line depicts a quantile regression fitted to the 90th percentile of the data, the dashed lines represent the polynomial logistic regression fit and the shaded area indicate the 95% confidence interval.

Fig. 4 presents the results of quantile regression applied to the natural log of the response ratio (RR) for grain yield (Fig. 4 a) and net marginal returns on fertilizer (Fig. 4 b) across different crops and regions under FFP. The RR tends to be lower when crop yield is high under FFP (Fig. 4 a), indicating that SSNM-DST is more beneficial for farmers with lower yields under their own practices. Both regression lines, one fitted to the data and another to the top 10% of data points, show an exponential decline in RR as the grain yield produced under farmers' own practices increases. For maize, the decline in RR is gradual for low-yielding farmers and steep for high-yielding ones. For rice and wheat, the decline in RR with increasing FFP yield is very steep from the beginning.

In Fig. 4 b, we observe that the impact of an increase in RR on net marginal returns (NMR) on fertilizer under SSNM practice varies depending on the crop and is mostly negative. For maize, an increase in RR leads to a linear decrease in NMR, which is surprising as we would usually expect returns to increase with higher response ratios. The trends for rice show no effect on NMR with increasing RR, and for wheat, the trends differ for low, medium, and high RR.

The results of a *meta*-regression analysis in Table 4 (Multiple R^2^ = 0.80, Adj. R^2^ = 0.77, F-statistic: 46.2 on 31 and 350 DF, p-value < 2.2e−16) indicate that the response ratio of using SSNM-DST over FFP is influenced by various non-tool related factors. These factors include crop yield under FFP, current and past crop types, soil acidity class, temperature and rainfall regimes, and the amount of input under FFP. For example, fields with higher yields under FFP tend to generate smaller RR when managed using SSNM-DST. A 15% increase in grain yield under FFP results in a statistically significant decline in RR when using SSNM-DST. The odds of a significant increase in the response ratio are approximately 1.5 times higher for maize, rice, and wheat compared to other crops when SSNM-DST is applied.

**Table 4:** Results of meta regression from modelling the effect of selected covariates on the magnitude of the response ratio on a logarithmic scale. Probabilities with significant effects are presented in bold. ε_i_ is the sampling error through which the effect size of a study deviates from its true effect, while ω_i_ is the true effect size of the study that is only sampled from an overarching distribution of effect sizes.

Variables*	Variable Category	Estimates	Std. Error ($\varepsilon_{i}$)	Statistic ($\omega_{i}$)	P values
FFP yield	Log (FFP) (kg/ha)	−0.15	0.02	−6.77	**0.0000**

*Current crop*					
	Maize	1.56	0.19	8.03	**0.0000**
	Rice	1.48	0.19	7.80	**0.0000**
	Wheat	1.47	0.18	8.02	**0.0000**

*pH class*					
	Slightly alkaline	−0.05	0.02	−2.58	**0.0104**
	Neutral	−0.03	0.02	−1.48	0.1385
	Slightly acidic	−0.05	0.01	−3.84	**0.0001**
	Acidic	−0.06	0.02	−3.47	**0.0006**
	Highly acidic	0.30	0.08	3.51	**0.0005**

*Precipitation regime*					
	Arid	0.16	0.05	2.99	**0.0030**
	Semi-arid	0.09	0.04	2.43	**0.0154**
	Sub-humid	0.03	0.03	0.88	0.3777
	Humid	0.02	0.03	0.66	0.5080
	Humid tropics	−0.12	0.07	−1.60	0.1108

*Temperature regime*					
	Sub-tropical	0.05	0.03	1.79	0.0750
	Tropical	−0.02	0.02	−0.63	0.5272
	Equatorial	0.05	0.03	1.52	0.1292

*Previous crop*					
	Maize	−0.05	0.05	−0.88	0.3796
	Legume	−0.21	0.07	−3.25	**0.0013**
	Rice	−0.09	0.05	−1.84	0.0671
	Wheat	−0.07	0.05	−1.27	0.2053

*Soil texture*					
	Clay	0.01	0.02	0.83	0.4059
	Loam	0.02	0.01	1.39	0.1669
	Sand	0.05	0.04	1.30	0.1933

*Inputs*					
	Average FFP P	0.01	0.00	2.68	**0.0076**
	Average FFP N	−0.01	0.00	−4.95	**0.0000**

* The variables used in this regression are described in https://doi.org/10.7910/DVN/IIONAF.

The response ratio is significantly more likely to be higher when SSNM-DST is applied on highly acidic soils, with a 30% increase in RR for such conditions. On the other hand, response ratios are likely to decrease significantly when the tools are applied on slightly alkaline soils. Application of SSNM-DST is also more likely to significantly increase the response ratio under drier conditions (arid and semi-arid) compared to wetter conditions, with odds ratios of 16% and 9%, respectively, for arid and semi-arid conditions. However, the use of SSNM-DST on fields with a legume as the preceding crop significantly reduces the response ratio. The odds that the RR declines significantly under such conditions is 21 %.

Interestingly, the application of higher rates of P fertilizer under FFP was found to increase the response ratio of using SSNM-DST significantly. Conversely, an increase of 1% in N fertilizer on FFP was found to reduce the response rate, which is expected as higher rates of fertilizer usually lead to increased yields under FFP, resulting in a reduced RR.

## 4 Discussion

### 4.1 Alternative SSNM-DST in smallholder farming systems have been expanding

In the 21st century, the widespread dominance of digital decision-support tools has significantly shaped various sectors across regions. In particular, we have observed a consistent increase in the development and promotion of digital and semi-digital tools in smallholder farming systems over the past three decades (Fig. 2). Previously, attempts were made to address the site-specificity of nutrient advisories through on-site diagnostics of plant characteristics (Thind and Gupta, 2010). It is important to note that soil heterogeneity, resulting from physical, chemical, and biological soil conditions, typically occurs at coarser scales (Goovaerts, 1998), although the applicability of such approaches beyond research settings has been contested due to documented soil heterogeneity over small spatial scales (Schut and Giller, 2020).

With the prevalence of mobile phones even in smallholder farming settings (CABI, 2019), digital tools based on PC, web, and app platforms began to emerge in the site-specific nutrient management advisory domain. This development initially took place in south Asia and southeast Asia (Chuan et al., 2013, Pampolino et al., 2012, Pooniya et al., 2015) and later expanding to SSA (Rurinda et al., 2020, Saito et al., 2015). These digital apps rely on observed field-specific variables, plot management history, and available inputs, which serve as proxies for soil fertility or nutrient responses. These factors play a crucial role in defining the application of these digital tools. Additionally, climate/weather information is an essential element in determining site specificity. Notably, only NE, the most popular SSNM-DST, employs climate information in generalized terms, while the other tools do not utilize such data (Fig. 2 and Table 1). It is worth mentioning that certain apps, such as FOT, make site-specific fertilizer use recommendations based on the concept of economic optimization, focusing on better resource allocation, rather than nutrient optimization for improved agronomic use efficiency.

### 4.2 Multiple factors limit application at scale of SSNM-DST in smallholder systems

Regardless of the increasing prevalence of SSNM-DST both in type and number (Fig. 2), the dominance of positive yields (Chivenge et al., 2021) and dominantly positive economic and environmental outcomes from application of the tools (Table 2), these tools have not been widely adopted (Fig. 3). The highest reported uptake of SSNM-DST by a target farmer community ranged between 20 % and 30 % (Fig. 3 a), and this was limited to just two advisory tools: NE and RCM. Similar findings were reflected in previous reports where the most adopted SSNM-DST, RCM, provided advise to about 30 % of the target farmers in the Philippines (Chivenge et al., 2021). Most of the expert respondents (65 %) estimated that the reach of these tools was either unknown or reached <5% of the target farming community. Despite minor variations among specific SSNM-DST, the overall scale of adoption has been low, with 54% of respondents indicating that these tools reached <1% of the target population of farmers (Fig. 3). Uptake of SSNM-DST has been minimal both for the private sector and government extension systems, suggesting a lack of interest from key stakeholders involved in scaling these technologies. Only 5–6 % of the experts reported full adoption of SSNM-DST by the private sector and government extension systems, while a substantial majority (72 – 76 %) stated that these nutrient management advisory tools have not been integrated into either the private business or government extension systems. Some experts reported partial uptake of these tools by these systems, but overall, the adoption rates for SSNM-DST are remarkably low compared to other digital technologies used by smallholders (Asravor et al., 2021) or similar digital decision support tools in large-scale agriculture (Kelley et al., 2020).

One of the main goals of this study was to investigate the factors influencing scaling in SSNM-DST. The survey of experts revealed that technical issues related to the tools were significant constraints for scaling these tools (Table 3), indicating that the tools are not technically ready for large-scale implementation. As shown in Table 3, technical, socio-economic, and policy constraints have varying effects on different groups within the user-chain of SSNM-DST. Technical challenges were found to be the most limiting factor for farmers and private businesses in adopting SSNM-DST at scale. The lack of involvement from private businesses in SSNM advisory tools suggests that these tools will struggle to reach scale through purely commercial means. Therefore, active public support is necessary as an initial prerequisite for these tools to establish themselves in smallholder systems. Despite the importance of public support for scaling SSNM-DST, our results highlight that the constraints to scale these tools are even more widespread for national extension systems (Table 3). Typically, only extension workers have access to the tools to generate recommendations. Given that extension workers need to cover numerous farmers and fields each season, this poses additional challenges for the widespread adoption of SSNM-DST (Andersson et al., 2020).

Our findings align with previous studies that have identified various barriers hindering the large-scale implementation of digital decision support tools in the smallholder context. These barriers include tools being too complex to use (Coggins et al., 2022), low levels of literacy and lack of skills (Chandra and Collis, 2021), limited access to smartphones or other electronic devices (Capalbo et al., 2018), inadequate assimilation of timely and relevant agronomic information (CABI, 2019) and poor integration with financial and input supply services (Vorotnikov et al., 2020). These findings suggest that SSNM-DST have been designed, developed, and deployed without adequately considering the needs and contexts of end users, primarily impoverished rural farming households, in terms of adoption and optimal implementation. It can be argued that these tools are still in their early stages of development and have the potential to mature in the future (Altunok and Cakmak, 2010). However, for such optimistic possibilities to become a reality, the demonstration, implementation, and promotion of these advisory tools should extend beyond experimental sites and selected ‘client’ farmers, as well as beyond project implementation periods. Complementing our results from the expert survey, detailed metanalysis of reported benefits of SSNM-DST (Table 4) reveal that non-tool-related covariates also determine the magnitude of response ratios. These differential responses arising from uncontrolled field, management, and environmental variations may contribute to the limited adoption of SSNM-DST.

### 4.3 Should site-specificity be a sufficient criterion for the success of SSNM-DST?

The results presented in Fig. 4 demonstrate that the yield advantages claimed for the use of SSNM-DST were not consistent across fields with varying initial productivity potentials. While the average yields may improve with the use of these tools, individual farmers experienced mixed results. Specifically, low-producing farmers benefited more from the tools compared to those who already had higher yields through their existing practices (Fig. 4 a). There could be several reasons for these findings. Firstly, low-producing farmers may have been using sub-optimal fertilizer and field management techniques, which the tools help address. In such cases, adopting the tools can lead to significant improvements in both yields and returns. Secondly, high-yielding farmers may already be employing optimal fertilizer rates and effective field management practices, reducing the potential for further gains through the advice provided by the tools. Thirdly, farmers may tend to apply excessive fertilizer rates as a precautionary measure to mitigate the risk of crop losses when expecting high yields. These factors highlight that developing the tool in a site-specific manner alone may not be sufficient for widespread adoption among farmers with varying initial production potentials. Andersson et al. (2020) propose considering an 'investment-based' advisory approach to address such heterogeneities.

This could create a situation where high-producing farmers, who gain relatively less from the advice, are less inclined to adopt SSNM-DST. On the other hand, low-producing farmers, who stand to benefit the most, may be constrained by limited resources, reducing their capacity to adopt the tool. This suggests that the farmers who would benefit the most from the advice are least likely to implement the tools due to resource constraints, while those who are more resource-endowed are less likely to adopt them as they stand to gain less from the advisories. Therefore, the context-specificity of SSNM-DST becomes essential in addition to their site-specificity. Advice provision should target farmers and fields based on their production potential, emphasizing the need for SSNM-DST to provide options rather than rigid, one-size-fits-all advice. Balancing the trade-off between resource constraints and the necessity of tool-assisted advice is crucial, at least, until the tools are scaled across the majority of the system.

Moreover, SSNM-DST predominantly focuses on tailoring advice to heterogeneous biophysical conditions while neglecting heterogeneities among users. This can potentially leave out the poor and vulnerable farmers living in marginal places, and women farmers, reminiscent of the pitfalls in traditional extension systems (Fabregas et al., 2019). This can also raise the question of “advice for whom?” If the advice is primarily intended for extension agents who then convey it to smallholder farmers, we encounter the issue of limited coverage. In many countries where smallholder farming is predominant, the ratio of farmers to extension agents is already high (Davis et al., 2010). Under such circumstances, the use of SSNM-DST becomes more challenging as it stretches the already overburdened extension workers with the task of generating and providing site-specific advisory services. Multiple site-specific advice is required even for a single farm, further hindering the possibility of scaling up these tools. In essence, the SSNM-DST have moved one step ahead compared with the traditional ‘blanket’ fertilizer recommendation by attuning to in-field heterogeneities in soil and crop characteristics, while remaining ‘blanket’ in terms of addressing demographic and socioeconomic heterogeneities among users, which potentially thwart adoption of the tools at scale.

### 4.4 Limitations of the study

Although this work tries to explore the potential and challenges in scaling SSNM-DST, some limitations were inevitable. First, the performance of the SSNM-DST appraised here were taken from the claims reported mainly from tool developers. We did not perform any independent performance analysis. This itself was only done from the user perspective. Second, detailed technical appraisal requires exclusive access to the algorithms behind the tools, which is lacking under the current appraisal. Third, given the heterogeneities in the principles behind each of the tools, specific constraints will also vary. The current analysis focuses on aggregate level constraints, with less emphasis on tool-dependent constraints of scaling. Fourth, we could not include the experiences and viewpoints of stakeholders (e.g., extension, policy makers) who have been implementing these tools because of logistics and practical issues. Fifth, the current appraisal could not allow for case-by-case and country-specific variables potentially constraining the scaling of the SSNM-DST, although Jacobs et al. (2018) identify the majority of the bottlenecks in technology scaling to be institutional by nature. This is important because scaling of SSNM-DST can be constrained by enabling environments (i.e., in addition to technical issues), which are highly influenced by country/regional institutional and governance setups. Future appraisals that include those institutional, socio-economic and governance aspects may improve our outlooks.

## 5 Conclusions

We have utilized a survey-*meta*-analysis hybrid methodology and systematically appraised the extent of and challenges to scaling decision support tools used for site-specific soil nutrient management in smallholder farming systems. Numerous such tools have been under promotion, and their application has been consistently rising, which is encouraging evidence that the concept of tool-assisted site-specific nutrient management has moved a long way from concept to practice. There is indication that SSNM-DST can improve crop productivity and economic return in smallholder production systems, although the scalability of these tools still needs extensive improvement. Application of these tools has been limited to ‘project life cycles’ mainly on experimental stations of research institutions and on farms of select ‘client’ smallholder farmers.

This appraisal has clearly revealed that practical issues, including tool complexity and heterogeneous benefits across users, are important tool- and non-tool-related variables with the potential of limiting scalability. While addressing these challenges can improve the potential scalability of SSNM-DST, future appraisals that include institutional, socio-economic, and governance aspects are needed to fully understand and address their current low scalability.

Capabilities that enable the tools to store data from previous advice and link it to subsequent advice, which are lacking in all tools appraised in the current work, can gradually enable the tools to serve as a source of data for the next advice. Although some of the tools (such as NE) can store data, the data are stored in formats that are not accessible by the tool during successive decisions. Such capabilities, where information flow becomes multidirectional between farmers and advice providers, assist easy knowledge exchange, documentation, and feedback. These data could be used for other purposes, especially if they could be stored on cloud-based servers where tool developers and other stakeholders can access them. Additionally, capabilities that allow access to legacy big data sources (such as soil maps, slope, climate, and land use maps) that could potentially be used to make decisions site-specific, are also lacking.

While our appraisal primarily focuses on the benefits of SSNM-DST for higher crop yields and greater economic returns, it is important to note that profitability can be improved even with lower yields if the overall profit is higher. Producers may prioritize profitability over higher yields and, therefore, it is essential to consider this aspect in assessing the success and desirability of decision support tools. In addition, our study also underscores the importance of addressing technical challenges, securing public support, and considering the specific needs and contexts of end users in order to successfully scale SSNM-DST. Additionally, the impact of non-tool-related factors on the adoption of these tools should be considered. This holistic approach will provide a comprehensive understanding of the impact and effectiveness of site-specific nutrient management tools in supporting sustainable agricultural practices and improving the livelihoods of smallholder farmers.

### CRediT authorship contribution statement

**Tesfaye Shiferaw Sida:** Conceptualization, Methodology, Data curation, Software, Visualization. **Samuel Gameda:** Conceptualization, Methodology, Supervision, Validation. **Jordan Chamberlin:** Conceptualization, Methodology, Supervision, Validation. **Jens A. Andersson:** Supervision, Validation. **Mezegebu Getnet Debas:** Conceptualization, Methodology, Supervision. **Lennart Woltering:** Methodology, Supervision, Validation. **Peter Craufurd:** Conceptualization, Methodology, Supervision, Validation.

## Declaration of Competing Interest

The authors declare that they have no known competing financial interests or personal relationships that could have appeared to influence the work reported in this paper.

## Data Availability

Data will be made available on request.
